# Transforaminal Endoscopic Decompression of Facet Cysts by Interventional Pain Physicians

**DOI:** 10.7759/cureus.18308

**Published:** 2021-09-27

**Authors:** Holden L Brown, Sanjeev Kumar

**Affiliations:** 1 Anesthesiology, University of Florida College of Medicine, Gainesville, USA

**Keywords:** surgical decompression, synovial cyst, spinal stenosis, endoscopic spine surgery, back pain

## Abstract

Lumbar synovial cysts (LSC) can impinge neural structures, causing radicular back pain. Conservative treatment options; however, are often ineffective, and traditional surgical techniques can cause joint instability. We describe two cases in which interventional pain physicians used transforaminal endoscopic spine surgery to treat LSC. The patients reported complete resolution of their lower back and radicular pain and the procedures preserved their motor function and sensation in their bilateral lower extremities. This technique is a viable option for remediation of LSC and can be performed by well-trained pain physicians.

## Introduction

Lumbar synovial cysts (LSC) are relatively uncommon, occurring in up to 0.5% of the general population, most often in the sixth decade of life and with a slight female preponderance [1]. They are often found incidentally, but occasionally increasing growth of the cyst into the spinal canal can impinge neural structures, often causing radicular back pain due to mechanical compression of the nerve/s. The cysts can vary in size from just a small simple indentation in the spinal canal to large complex cysts extending several segments. The lower lumbar spine is usually the most common site for these cysts and it can form due to repetitive mechanical stress at the facet joint. Conservative therapy includes image-guided cyst drainage and/or steroid injections [2,3]. These conservative options are often of limited efficacy in the treatment of LSC and patients are commonly referred for surgical evaluation. Traditional surgical techniques include decompressive laminectomy, medial facetectomy, and cyst excision; however, this invasive approach can predispose patients to further joint instability [4,5]. Spine surgeons have used endoscopic decompression to treat various pathologies of the lumbar spine. The safety profile of this procedure has been established [6], and it has shown favorable outcomes with durable pain relief [7]. Transforaminal endoscopic spine surgery is an emerging technique for the treatment of LSC. Well-trained pain physicians have used the procedure to relieve radicular pain associated with lumbar spinal stenosis. Although the authors are aware of several successful endoscopic facet cyst decompression surgeries conducted by interventional pain physicians, to our knowledge these are the first two reported cases of facet cyst endoscopic decompression performed in an academic pain medicine program in the United States.

## Case presentation

Case 1

**Figure 1:**
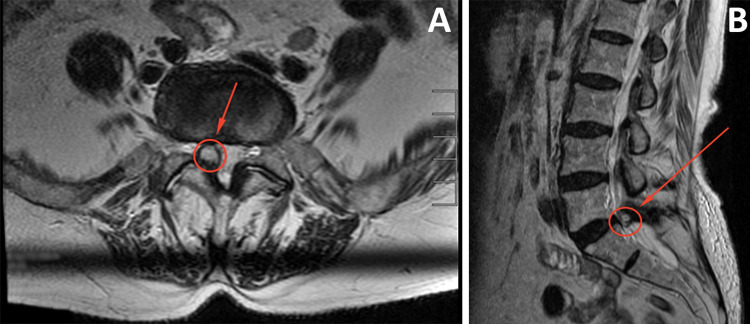
(A) Preoperative axial view of the facet cyst indicated by the circle and arrow in T2 as seen on magnetic resonance imaging. (B) Sagittal view of the cyst. The patient’s significant radicular symptoms can be correlated with the stenosis caused by the cyst.

An 87-year-old woman presented with persistent lumbar radicular pain that had been present for 2.5 months. The pain was in her right lower back and radiated down the back of her leg in the L5-S1 distribution. There were no inciting events. The patient had limited improvement in symptoms with two rounds of methylprednisolone, acetaminophen, physical therapy, nonsteroidal anti-inflammatory drugs, and tramadol. She experienced some relief with oxycodone. Magnetic resonance imaging (MRI) showed a right L5-S1 facet cyst compressing the dural sac, as well as traversing the S1 nerve root (Figures 1A, 1B).

This patient experienced complete resolution of her symptoms in the immediate postoperative period on the day of the procedure, which continued until postoperative day 4. The patient lives out of state and returned to her home after the one-week postoperative check. She was instructed to contact the clinic if her pain returned. There was no follow-up directly with her since the surgery due to the distance involved but a close family member who is also a patient in our practice gave an update on her well-being and lack of any symptoms about one year after her surgery.

Case 2

**Figure 2:**
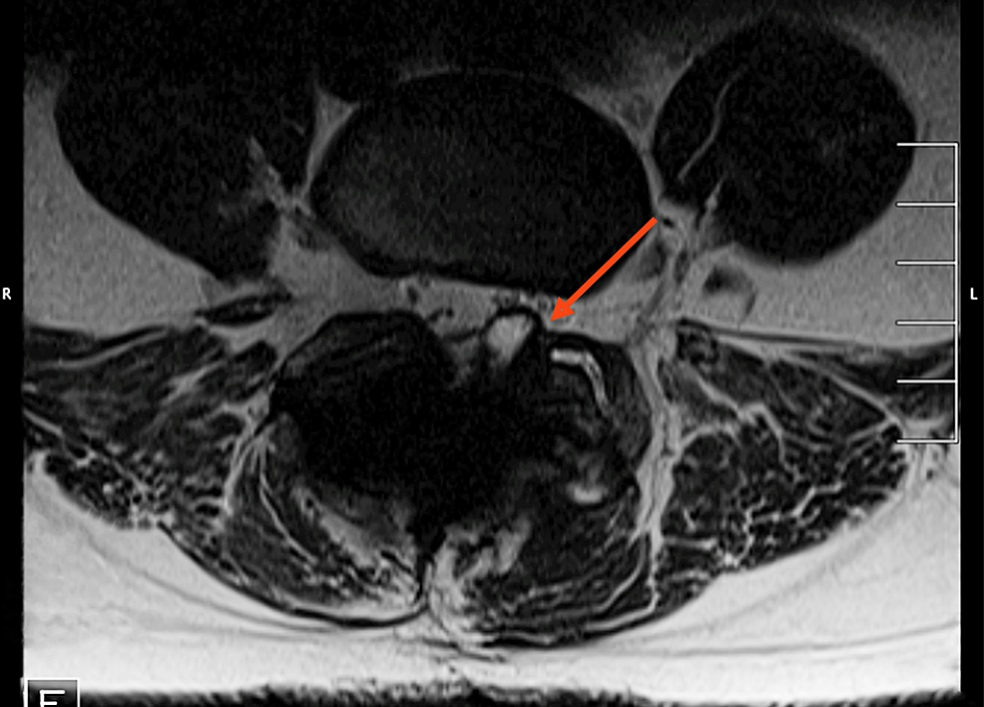
Preoperative axial cross-sectional T2 magnetic resonance imaging of the L4-L5 level. The facet cyst is visible on the left facet joint.

A 54-year-old woman presented with back pain and radicular pain in her left leg with a mild sensory deficit but no motor weakness. Her back pain came on suddenly after a bicycle accident 4 months prior. An MRI revealed a 1.3-×-0.7-cm LSC extending medially from the left L4-L5 facet joint, combined with an asymmetrical left disc bulge causing mass effect and nerve impingement of the transiting L5 and likely S1 nerve roots (Figure 2). Conservative therapies including physical therapy, chiropractic therapy, massage therapy, celecoxib, gabapentin, and yoga were attempted with limited success.

**Figure 3:**
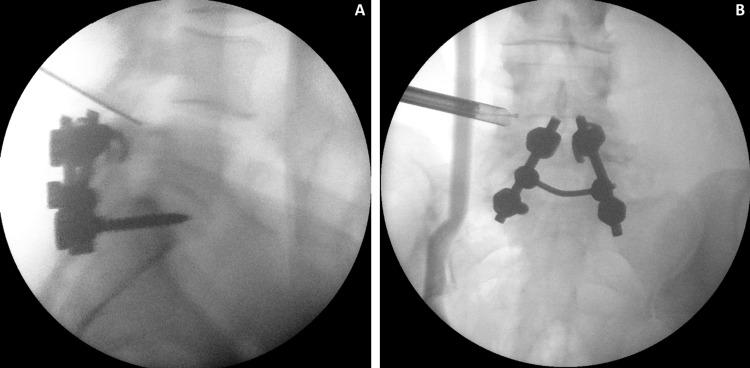
(A) Lateral view of the lumbar spine with the indicator pointed at the target pathology. (B) The anteroposterior view shows the endoscope in place near the facet cyst.

**Figure 4:**
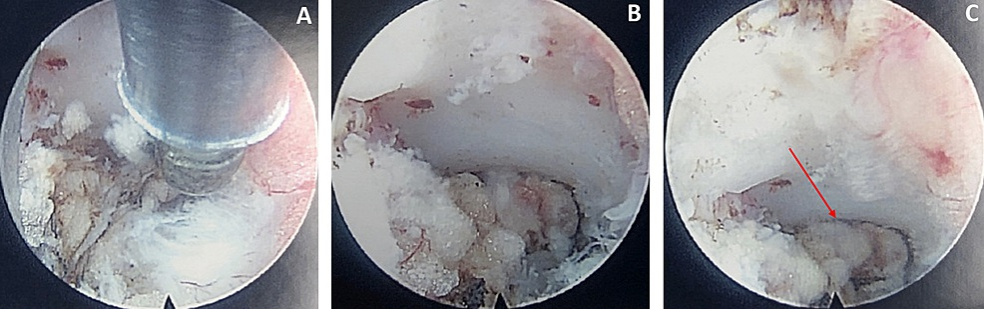
(A-C) Endoscopic camera views of the left L4-L5 facet joint (chronological order). Progressive debulking of the facet pathology can be seen. The red arrow indicates the fenestrated cyst wall after the cystic fluid has been drained from it.

The patient reported immediate resolution of her radicular symptoms after the procedure. Figures 3A, 3B, 4A-4C depict the pathology intraoperatively. She followed up by phone on postoperative day 1 and in the clinic on postoperative day 4. She reported complete resolution of her radicular leg pain and endorsed only slight surgical site pain. A physical examination found that her deep tendon reflexes, strength, and sensation in her bilateral lower extremities were normal.

Transforaminal endoscopic operative technique

The procedures were performed under local anesthesia with mild intravenous sedation utilizing midazolam, dexmedetomidine, and remifentanil infusion. Sedation was titrated such that the patient and surgeon could communicate throughout the procedure. A 16-gauge needle was introduced to target the inferior part of the neuroforamen using fluoroscopic guidance. Once placement was deemed satisfactory, a guidewire was placed through the needle and the needle was removed. At that point, a 1-cm skin incision was made and a fasciotomy was created. A series of serial dilators and trephines were introduced before the placement of a 7.2-mm beveled working tube, through which a 6.3-mm spinal endoscope was placed. Continuous normal saline irrigation was maintained throughout the procedure via the spinal endoscope. A Kerrison rongeur was inserted to create a partial facetectomy. The foraminal area was carefully dissected and the epidural space and facet cyst pathology were visualized. Using a combination of punch, pituitary forceps, and bipolar radiofrequency cautery, the cyst wall was fenestrated and the cyst material was removed. The traversing nerve root was visualized moving freely in the epidural space, signifying adequate decompression. These surgical steps were performed in both patients, who reported immediate improvement in their radicular symptoms at the end of the procedure.

Postoperative course

The surgeries were performed under local anesthesia with mild intravenous sedation. The surgical team could examine the patient’s neurological function in the operating room during the procedure. The patients reported complete resolution of lower back and radicular pain. The patients’ motor function and bilateral lower extremity sensation were preserved on their postoperative follow-up visits.

## Discussion

Back pain is a debilitating condition that affects almost 13% of the adult US population between 20 and 69 years old at any given point. It leads to the suffering of the affected individual, as well as broader economic and healthcare burdens. Conservative treatments for back pain include rest, nonsteroidal anti-inflammatory drugs, and physical therapy; however, if these prove ineffective, then more invasive treatments are sometimes warranted. Interventional pain physicians have a number of modalities that often prove effective at alleviating back pain, ranging from medication optimization to various injections and nerve ablations, among others. The choice of intervention depends on a comprehensive assessment by patient history, physical examination, and imaging studies. Some pathologies, however, have not been treatable with interventions offered by pain physicians; one such pathology is LSC. These have previously been relegated to treatment by traditional spine surgery.

These two cases describe a relatively new approach to treating a problem that was traditionally managed surgically. The surgical approach, while effective, is invasive and contributes to facet joint destabilization. This less-invasive technique, endoscopic decompression, leads to greater preservation of bony structure and muscles, minimal epidural fibrosis or scarring, and faster recovery times [6-8]. Literature supports endoscopic decompression as an acceptable strategy for treating LSC [4,5].

It is also important to note that with proper training, interventional pain physicians can safely and effectively perform endoscopic spine surgery. The endoscopic approach can be used for treating various pathologies of the lumbar spine such as lumbar disc herniation, lumbar spinal stenosis, and lumbar epidural fibrosis/scarring from the lumbar post-laminectomy syndrome. This procedure offers interventional pain physicians a safe and effective option to treat previously recalcitrant pain conditions and provides patients a safe alternative to traditional spine surgery.

## Conclusions

These two patients reported complete resolution of lower back and radicular pain after undergoing transforaminal endoscopic decompression of facet cysts by interventional pain physicians. Transforaminal endoscopic surgery for symptomatic lumbar facet cyst causing radicular symptoms is an acceptable and even preferable approach to remediation of LSC. Properly trained pain physicians can use this technique.
